# Predictive factors of early outcome after palliative surgery for colorectal carcinoma

**DOI:** 10.1515/iss-2020-0018

**Published:** 2020-11-02

**Authors:** Ralf Konopke, Jörg Schubert, Oliver Stöltzing, Tina Thomas, Stephan Kersting, Axel Denz

**Affiliations:** Elblandklinikum Riesa, Zentrum für Allgemein- und Viszeralchirurgie Riesa-Meißen, Meissen, Germany; Elblandklinikum Riesa, Klinik für Innere Medizin II, Meissen, Germany; Universitätsklinikum Dresden, Medizinische Klinik I, Dresden, Germany; Universitätsmedizin Greifswald, Klinik und Poliklinik für Allgemeine Chirurgie, Viszeral-, Thorax- und Gefäßchirurgie, Greifswald, Germany; Chirurgische Klinik, Universitätsklinikum Erlangen, Erlangen, Germany

**Keywords:** colorectal cancer, metastasis, palliative surgery, palliative treatment, surgery

## Abstract

**Objectives:**

A significant number of patients with colorectal cancer are presented with various conditions requiring surgery in an oncologically palliative setting. We performed this study to identify risk factors for early outcome after surgery to facilitate the decision-making process for therapy in a palliative disease.

**Methods:**

We performed a retrospective chart review of 142 patients who underwent palliative surgery due to locally advanced, complicated, or advanced metastatic colorectal carcinoma between January 2010 and April 2018 at the “Elbland” Medical Center Riesa. We performed a logistic regression analysis of 43 factors to identify independent predictors for complications and mortality.

**Results:**

Surgery included resections with primary anastomosis (n=31; 21.8%) or discontinuous resections with colostomy (n=38; 26.8%), internal bypasses (n=27; 19.0%) and stoma formation only (n=46; 32.4%). The median length of hospitalization was 12 days (2–53 days), in-hospital morbidity was 50.0% and the mortality rate was 18.3%. Independent risk factors of in-hospital morbidity were age (HR: 1.5, p=0.046) and various comorbidities of the patients [obesity (HR: 1.8, p=0.036), renal failure (HR: 1.6, p=0.040), diabetes (HR: 1.6, p=0.032), alcohol abuse (HR: 1.3, p=0.023)] as well as lung metastases (HR: 1.6, p=0.041). Arteriosclerosis (HR: 1.4; p=0.045) and arterial hypertension (HR: 1.4, p=0.042) were independent risk factors for medical complications in multivariate analysis. None of the analyzed factors predicted the surgical morbidity after the palliative procedures. Emergency surgery (HR: 10.2, p=0.019), intestinal obstruction (HR: 9.2, p=0.006) and ascites (HR: 5.0, p=0.034) were multivariate significant parameters of in-hospital mortality.

**Conclusions:**

Palliatively treated patients with colorectal cancer undergoing surgery show high rates of morbidity and mortality after surgery. In this retrospective chart review, independent risk factors for morbidity and in-hospital mortality were identified that are similar to patients in curative care. An adequate selection of patients before palliative operation should lead to a better outcome after surgery. Especially in patients with intestinal obstruction and ascites scheduled for emergency surgery, every effort should be made to convey these patients to elective surgery by interventional therapy, such as a stent or minimally invasive stoma formation.

## Background

Despite recent advances in surgical and oncological therapies of colorectal carcinoma (CRC), in the end stage of their disease, most patients require palliative care. In palliative situations, surgical management requires a high level of expertise to obtain optimal results regarding the individual demands of the patient. Depending on the general condition of the patient, decisions on best supportive care, interventional or medical therapy, or a surgical procedure must be made individually. Further aspects are the patient’s comorbidities, local tumor status and existing metastasis, as well as the urgency of treatment. The decision on therapeutic strategy and the estimation of prognosis and possible outcome should ultimately be reached on a multidisciplinary basis after a detailed investigation of the patient and, if applicable, in discussion with his relatives.

Despite the high incidence of the CRC, the amount of data contributing to decision making in the palliative situation is minimal. Clinical studies to further evaluate the results of different palliative treatments and offer the patient a tailored therapeutical concept for his advanced malignant disease are needed.

This unicentric study aimed to analyze factors influencing the early postoperative outcome of patients after palliative surgery for colorectal cancer. All interventions were performed to avoid or treat complications of the tumor in the end-stage of the disease.

The ethics committee of the Technical University of Dresden positively evaluated the implementation of the study. We present the following article following the STROBE reporting checklist for observational studies.

## Methods

The medical data of 161 patients undergoing surgery for incurable colorectal carcinoma at Elblandklinikum Riesa in the period from 2010 to 2018 were retrospectively reviewed.

Cases with surgical exploration only (n=3), existing contradicting living will, and resulting therapy limitations (n=11), as well as those with incomplete data set (n=5), were excluded. After all, we included 142 patients in the final analysis.

### Preoperative strategy

The diagnostic assessment was tailored to the clinical condition of the patient. In addition to anamnesis and clinical examination, a comprehensive laboratory analysis with small blood count, C-reactive protein (CRP), coagulation analysis (Quick, International Normalized Ratio [INR], Partial Thromboplastin Time [PTT]), determination of electrolytes and urine retention parameters (Urea, creatinine and glomerular filtration rate) and liver-specific parameters (albumin, bilirubin, aspartate aminotransferase [ASAT], alanine aminotransferase [ALAT], gamma-glutamyltransferase [gamma-GT] and alkaline phosphatase [AP]) was obtained. At least one imaging procedure (X-ray, sonography or computed tomography of the abdomen) was used to assess the urgency of surgical measures and the extent of tumor disease.

Patients with related comorbidities received a chest X-ray and an ECG for anesthesiologic evaluation.

Concluding the available results, the urgency of the surgical intervention was determined.

### Intraoperative management

A total of 11 general or visceral surgeons with specialist standard performed the operations. A laparoscopic approach was preferred.

The extent of surgery was based on the intraoperative findings and the appropriateness of the procedure, considering the concomitant diseases and the current condition of the patient.

Resecting procedures were preferably performed as tubular and segmental resections striving for complete tumor removal without observing oncological principles such as radical lymphadenectomy.

When applying a bypass anastomosis, bowel segments located orally and aborally of the tumor were usually connected by a side-to-side-anastomosis. The creation of a double-barrel ostomy provided stool deviation orally from the carcinoma.

### Postoperative procedure

Primary extubation was always intended after surgery. Most of the patients were initially treated in the intensive care unit. Depending on their recovery, the treatment was continued on a standard-care surgical ward.

Routine postoperative examinations included an X-ray of the thorax on POD 1. Comprehensive laboratory analysis was performed to monitor anemia, coagulation disorders, renal and liver function and inflammatory changes on POD 1 and 2.

Further investigations were carried out in cases of clinical suspicion of complications. They included lab tests, sonography, computed tomography scan and endoscopic procedures.

Complications were defined as all events that had a decisive influence on the patient’s recovery and led to an extended stay in hospital and/or death. Infectious complications were diagnosed, in addition to imaging, by increased inflammation parameters (CRP, leukocytes, PCT) and related clinical symptoms.

Each death of a patient during the inpatient stay was assigned to the mortality rate, regardless of the period passed after palliative surgery for colorectal cancer.

### Follow-up

The follow-up ended on the day of discharge from the hospital and included a comprehensive clinical examination and a final laboratory screening similar to the investigation before surgery. In the case of existing complications, patients were repeatedly seen and checked as out-clinic patients.

### Statistics

All data were anonymized and transferred to the IBM SPSS Statistics 25 software.

For the following preoperative parameters, an analysis was performed with regard to differences between surgical procedures and their influence on morbidity and mortality after surgery: age, sex, concomitant diseases of the patient before the intervention (anemia, nicotine abuse, alcohol abuse, cachexia according to DGEM [1], diabetes mellitus, arteriosclerosis, previous deep vein thrombosis/embolism, chronic bronchitis/bronchial asthma, arterial hypertension, coronary heart disease [CHD], renal insufficiency from stage II after KDIGO [2], hyperalbuminemia, hyperbilirubinemia, liver scarring/cirrhosis, obesity, anticoagulation, cerebral morbidity, ascites, ASA score), tumor-associated symptoms (unintended weight loss of at least 10% within 6 months, pain according to numerical rating scale [3]), characteristics of the carcinoma (locally advanced tumor, metastasis pattern, localization), time between initial diagnosis of the tumor disease and surgical intervention, previous chemo and/or radiotherapy, indication for surgery (ileus, subileus, tumor bleeding, tumor perforation, asymptomatic) as well as time interval between presentation of the patient until surgery.

Intra- and postoperative factors (duration of surgery, perioperative blood loss, the substitution of erythrocyte concentrates and coagulation-active plasma in an interval up to 48 h postoperatively) were compared between the procedures.

We used Fisher’s exact test, Pearson’s chi-square test (dichotomous variables) and Student’s *t*-test (metric variables) for calculations of existing correlations between groups with different surgical procedures and univariate analyses on the influence on morbidity and mortality after surgery.

For multivariate analysis to demonstrate an independent influence of preoperative factors on postoperative complications and mortality, we used linear and binary logistic regressions.

In all statistical evaluations, a p-value of less than 0.05 was considered significant.

## Results

### OP procedure

In 142 patients, the carcinoma was resected with primary anastomosis in 31 cases (21.8%) and discontinuity resection with blind closure of the aboral bowel and terminal stoma in 38 cases (26.8%). Twenty-seven patients received an internal bypass anastomosis (19.0%), and 46 times a double-barrel stoma (32.4%) was created. Palliative abdomino-perineal rectal extirpations were not performed in the patient cohort.

The bypass anastomoses were performed as ileotransversostomy in 16 cases, as ileosigmoideostomy in 4 patients, as transversosigmoidestomy in 3 patients and as ileorectostomy in 4 cases.

In 26 cases, the double-barrel stoma was applied in the section of the sigmoid, eight times in the transverse colon, and in 12 patients in the ileum.

A total of 52 operations (36.6%) were done laparoscopically.

### Demographic data and patient comorbidity

Table 1 shows the results of the univariate comparative analysis between palliative surgical procedures for colorectal cancer with regard to demography and comorbidity of the patients.

**Table 1:** Demographic data and comorbidity of 142 patients with colorectal cancer in univariate comparison of palliative surgical procedures.

Parameters	Total n (%)	Resection with anastomosis (n=31) n (%)	Discontinuity resection (n=38) n (%)	Internal bypass anastomosis (n=27) n (%)	Ostomy formation (n=46) n (%)	p-Value
*Demographic data*						
Gender						
male	81 (57.0)	18 (22.2)	19 (23.5)	12 (14.8)	32 (39.5)	0.140
Female	61 (43.0)	13 (21.3)	19 (31.3)	15 (24.6)	14 (23.0)	
Age						
<75 years	69 (48.6)	15 (21.7)	15 (21.7)	15 (21.7)	24 (34.8)	0.567
>75 years	73 (51.4)	16 (21.9)	23 (31.5)	12 (16.4)	22 (30.1)	
*Co-morbidity*						
Anemia						
Yes	82 (57.7)	16 (19.5)	18 (22.0)	18 (22.0)	30 (36.5)	0.252
No	60 (42.3)	15 (25.0)	20 (33.3)	9 (15.0)	16 (26.7)	
Nicotine abuse						
Yes	15 (10.7)	3 (20.0)	3 (20.0)	3 (20.0)	6 (40.0)	0.829
No	127 (89.3)	28 (22.0)	35 (27.6)	24 (18.9)	40 (31.5)	
Alcohol abuse						
Yes	10 (7.0)	2 (20.0)	4 (40.0)	0	4 (40.0)	0.398
No	132 (93.0)	29 (22.0)	34 (25.8)	27 (20.4)	42 (31.8)	
Cachexia [1]						
Yes	42 (29.6)	2 (4.8)	6 (14.3)	9 (21.4)	25 (59.5)	0.001
No	100 (70.4)	29 (29.0)	32 (32.0)	18 (18.0)	21 (21.0)	
Diabetes mellitus type II						
Yes	42 (29.6)	7 (16.7)	14 (33.3)	9 (21.4)	12 (28.6)	0.543
No	100 (70.4)	24 (24.0)	24 (24.0)	18 (18.0)	34 (34.0)	
Arteriosclerosis						
Yes	24 (16.9)	3 (12.5)	6 (25.0)	9 (37.5)	6 (25.0)	0.076
No	118 (83.1)	28 (23.7)	32 (27.1)	18 (15.3)	40 (33.9)	
DVT/Embolism anamnestic						
Yes	20 (14.1)	5 (25.0)	4 (20.0)	3 (15.0)	8 (40.0)	0.773
No	122 (85.9)	26 (21.3)	34 (27.9)	24 (19.7)	38 (31.1)	
Chronic bronchitis/asthma bronchial						
Yes	11 (7.7)	2 (18.2)	3 (27.3)	6 (54.5)	0	0.008
No	131 (92.3)	29 (22.2)	35 (26.7)	21 (16.0)	46 (35.1)	
*Co-morbidity*						
Arterial hypertension						
Yes	91 (64.1)	22 (24.2)	27 (29.7)	18 (19.8)	24 (26.3)	0.226
No	51 (35.9)	9 (17.6)	11 (21.6)	9 (17.6)	22 (43.2)	
CHD						
Yes	29 (20.4)	7 (24.1)	7 (24.1)	0	15 (51.8)	0.010
No	113 (79.6)	24 (21.3)	31 (27.4)	27 (23.9)	31 (27.4)	
Renal insufficiency[2]						
≥Grade 2	39 (27.5)	7 (17.9)	13 (33.3)	6 (15.5)	13 (33.3)	0.653
≤Grade 1	103 (72.5)	24 (23.3)	25 (24.3)	21 (20.4)	33 (32.0)	
Hypalbuminemia						
>3,5 g/dL	41 (28.9)	5 (12.3)	6 (14.6)	6 (14.6)	24 (58.5)	0.001
<3.5 g/dL	101 (71.1)	26 (25.7)	32 (31.7)	21 (20.8)	22 (21.8)	
Hyperbilirubinemia						
> 20,5 µmol/L	16 (11.3)	1 (6.3)	4 (25.0)	9 (56.3)	2 (12.4)	0.001
<20,5 µmol/L	126 (88.7)	30 (23.8)	34 (27.0)	18 (14.3)	44 (34.9)	
Liver remodeling /Cirrhosis						
Yes	30 (21.1)	4 (13.3)	9 (30.0)	6 (20.0)	11 (36.7)	0.650
No	112 (78.9)	27 (24.1)	29 (25.9)	21 (18.8)	35 (31.2)	
Obesity						
>30 kg/m^2^	18 (12.7)	3 (16.7)	8 (44.4)	3 (16.7)	4 (22.2)	0.337
<30 kg/m ^2^	124 (87.3)	28 (22.6)	30 (24.2)	24 (19.4)	42 (33.8)	
Anti-coagulation						
Yes	27 (19.0)	8 (29.6)	5 (18.5)	6 (22.3)	8 (29.6)	0.565
No	115 (81.0)	23 (20.0)	33 (28.7)	21 (18.3)	38 (33.0)	
Cerebral insufficiency						
Yes	16 (11.3)	5 (31.3)	9 (56.3)	0	2 (12.4)	0.007
No	126 (88.7)	26 (20.6)	29 (23.1)	27 (21.4)	44 (34.9)	
Ascites						
Yes	60 (42.3)	10 (16.7)	10 (16.7)	15 (25.0)	25 (41.6)	0.019
No	82 (57.7)	21 (25.6)	28 (34.1)	12 (14.7)	21 (25.6)	
ASA score						
1	1 (0.70)	1 (100)	0	0	0	0.002
2	66 (46.5)	21 (31.8)	22 (33.3)	12 (18.2)	11 (16.7)	
3	68 (47.9)	9 (13.2)	16 (23.5)	12 (17.6)	31 (45.7)	
4	7 (4.9)	0	0	3 (42.9)	4 (57.1)	

DVT, Deep venous thrombosis; CHD, Coronary heart disease.

Significant but more limited operations (ostomy or internal bypass anastomosis) were performed in case of preexisting cachexia, chronic bronchitis or bronchial asthma, coronary heart disease, hypalbuminemia, hyperbilirubinemia, ascites as well as high ASA stratification (3 or 4). In the absence of comorbidities (ASA 1 and 2) and in cases with only cerebral comorbidity, mainly resecting procedures were carried out.

### Tumor-associated symptoms and characteristics, multimodal treatment concepts

Among the analyzed tumor-associated symptoms, a preoperative relevant reduction of body mass resulted significantly more often in limited operations. In contrast, a stable body weight resulted in more resections but also in the creation of a stoma (see Table 2).

**Table 2:** Univariate comparison of symptoms and characteristics of colorectal carcinomas and oncological therapies in 142 palliative interventions.

Parameters	Total n (%)	Resection with anastomosis (n=31) n (%)	Discontinuity resection (n=38) n (%)	Internal bypass anastomosis (n=27) n (%)	Ostomy formation (n=46) n (%)	p-Value
*Tumor-associated symptoms*						
Weight-loss						
Yes	46 (32.4)	8 (17.4)	11 (23.9)	15 (32.6)	12 (26.1)	0.041
No	96 (67.6)	23 (24.0)	27 (28.1)	12 (12.5)	34 (35.4)	
Pain [3]						
NRS 6–10	29 (20.4)	3 (10.3)	8 (27.6)	6 (20.7)	12 (41.4)	0.366
NRS 0–5	113 (79.6)	28 (24.8)	30 (26.5)	21 (18.6)	34 (30.1)	
*Tumor characteristics*						
Tumor local advanced						
Yes	132 (93.0)	28 (21.2)	35 (26.5)	27 (20.5)	42 (31.8)	0.456
No	10 (7.0)	3 (30.0)	3 (30.0)	0	4 (40.0)	
*Tumor localization*						
Right colon						
Yes	40 (28.2)	14 (35.0)	2 (5.0)	16 (40.0)	8 (20.0)	0.010
No	102 (71.8)	17 (16.7)	36 (35.3)	11 (10.8)	38 (37.2)	
Left colon						
Yes	18 (12.7)	6 (33.3)	3 (16.7)	7 (38.9)	2 (11.1)	0.022
No	124 (87.3)	25 (20.2)	35 (28.2)	20 (16.1)	44 (35.5)	
Sigma						
Yes	30 (21.1)	4 (13.3)	12 (40.1)	4 (13.3)	10 (33.3)	0.058
No	112 (78.9)	27 (24.1)	26 (23.2)	23 (20.5)	36 (32.2)	
Rectum						
Yes	54 (38.0)	7 (13.0)	21 (38.9)	0	26 (48.1)	0.019
No	88 (62.0)	24 (27.3)	17 (19.3)	27 (30.7)	20 (22.7)	
*Metastases*						
Liver						
Yes	72 (50.7)	17 (23.6)	19 (26.4)	12 (16.7)	24 (33.3)	0.877
No	70 (49.3)	14 (20.0)	19 (27.1)	15 (21.4)	22 (31.5)	
Lungs						
Yes	29 (20.4)	8 (27.6)	8 (27.6)	3 (10.3)	10 (34.5)	0.526
No	113 (79.6)	23 (20.4)	30 (26.5)	24 (21.2)	36 (31.9)	
Peritoneum						
Yes	66 (46.5)	12 (18.2)	19 (28.8)	15 (22.7)	20 (30.3)	0.527
No	76 (53.5)	19 (25.0)	19 (25.0)	12 (15.8)	26 (34.2)	
*Chronological trend*						
Initial diagnosis until OP						
<12 months	6 (4.2)	1 (16.7)	0	3 (50.0)	2 (33.3)	0.178
>12 months	136 (95.8)	30 (22.1)	38 (27.9)	24 (17.6)	44 (32.4)	
*Multimodal therapy*						
Chemotherapy						
≤6 weeks	12 (24.3)	3 (25.0)	7 (58.3)	0	2 (16.7)	0.162
>6 weeks	100 (75.7)	23 (23.0)	26 (26.0)	22 (22.0)	29 (29.0)	
Radio						
≤6 weeks	6 (15.0)	1 (16.7)	5 (83.3)	0	0	0.154
>6 weeks	32 (85.0)	9 (28.1)	10 (31.3)	0	13 (40.6)	

Regarding tumor characteristics, a similar distribution of interventions was found for tumors in the right and left colons, with a significant accumulation of resections with primary anastomosis and the creation of an internal bypass. Rectal malignancies were mainly treated with a stoma formation, less frequently with resection in discontinuity.

The detection and localization of metastases, the time between the initial diagnosis of the malignancy and the intervention, and previous chemo- or radiotherapy within a 6-week interval did not have a significant influence on the choice of surgical procedure.

### Indication and urgency

Concerning the indication for surgery, only tumor perforation influenced the choice of surgery (see Table 3). The patients received significantly more often a stoma.

**Table 3:** Univariate comparative analysis of palliative surgical procedures with regard to indication and urgency of intervention in 142 patients with colorectal cancer.

Parameters	Total n (%)	Resection with anastomosis (n=31) n (%)	Discontinuity resection (n=38) n (%)	Internal bypass anastomosis (n=27) n (%)	Ostomy formation (n=46) n (%)	p-Value
*OP indication*						
Tumor asymptomatic						
Yes	14 (9.9)	6 (42.9)	3 (21.4)	3 (21.4)	2 (14.3)	0.177
No	128 (90.1)	25 (19.5)	35 (27.3)	24 (18.8)	44 (34.4)	
Subileus						
Yes	41 (28.9)	4 (9.8)	13 (31.7)	6 (14.6)	18 (43.9)	0.063
No	101 (71.1)	27 (26.7)	25 (24.8)	21 (20.8)	28 (27.7)	
Ileus						
Yes	60 (42.3)	13 (21.7)	16 (26.7)	15 (25.0)	16 (26.7)	0.390
No	82 (57.7)	18 (22.0)	22 (26.8)	12 (14.6)	30 (36.6)	
Tumor bleeding acute						
Yes	3 (2.0)	2 (66.7)	1 (33.3)	0	0	0.217
No	139 (98.0)	29 (20.9)	37 (26.6)	27 (19.4)	46 (33.1)	
Tumor bleeding subacute						
Yes	0	0	0	0	0	0.148
No	142 (100)	31 (21.8)	38 (26.8)	27 (19.0)	46 (32.4)	
Tumor bleeding chronic						
Yes	10 (7.0)	4 (40.0)	3 (30.0)	3 (30.0)	0	0.120
No	132 (93.0)	27 (20.5)	35 (26.5)	24 (18.2)	46 (34.8)	
Tumor-perforation						
Yes	14 (9.9)	2 (14.3)	2 (14.3)	0	10 (71.4)	0.009
No	128 (90.1)	29 (22.7)	36 (28.1)	27 (19.0)	36 (25.4)	
*Urgency OP*						
Most urgent (≤6 h)	79 (55.6)	10 (13.7)	20 (25.3)	18 (22.8)	31 (39.2)	0.012
Urgent/elective (>6 h)	63 (44.4)	21 (33.3)	18 (28.6)	9 (14.3)	15 (23.8)	

Urgent surgery within 6 h after the presentation of the patient mostly allowed only for the creation of a stoma. In contrast, in elective surgery, more than 50% of the operations were completed with the removal of the tumor.

### Perioperative factors

Resection procedures showed significantly longer surgery times, higher perioperative blood loss and increased consumption of red cell concentrates (see Table 4).

**Table 4:** Perioperative parameters in univariate comparison of palliative surgical procedure in 142 patients with colorectal cancer.

Parameters	Total n (%)	Resection with anastomosis (n=31) n (%)	Discontinuity resection (n=38) n (%)	Internal bypass anastomosis (n=27) n (%)	Ostomy formation (n=46) n (%)	p-Value
Operating time						
≥130 min	78 (54.9)	24 (30.8)	34 (43.6)	6 (7.7)	14 (17.9)	
<130 min	64 (45.1)	7 (10.9)	4 (6.3)	21 (32.8)	32 (50.0)	0.001
Perioperative blood loss						
≥200 mL	63 (44.4)	24 (38.1)	33 (52.4)	0	6 (9.5)	
<200 mL	79 (55.6)	7 (8.9)	5 (6.3)	27 (34.2)	40 (50.6)	0.001
Erythrocytes concentrates (until 48 h postop.)						
Yes	36 (25.4)	11 (30.6)	14 (38.9)	3 (8.3)	8 (22.2)	
No	106 (74.6)	20 (18.9)	24 (22.6)	24 (22.6)	38 (35.9)	0.033
Administration of fresh frozen plasma (until 48 h postop.)						
Yes	25 (17.6)	5 (20.0)	9 (36.0)	3 (12.0)	8 (32.0)	
No	117 (82.4)	26 (22.2)	29 (24.8)	24 (20.5)	38 (32.5)	0.615

### Morbidity and mortality

The overall morbidity rate was 50.0% (see Table 5). 25.4% of patients had one, 12.4% two, 6.1% three and 6.1% more than three complications.

**Table 5:** Postoperative complications and mortality in 142 palliative surgeries for colorectal cancer, univariate analysis of the frequency of the performed procedures.

Parameters	Total n (%)	Resection with anastomosis (n=31) n (%)	Discontinuity resection (n=38) n (%)	Internal bypass anastomosis (n=27) n (%)	Ostomy formation (n=46) n (%)	p-Value
*Morbidity total*						
Yes	71 (50.0)	18 (25.4)	14 (19.7)	14 (19.7)	25 (35.2)	0.281
No	71 (50.0)	13 (18.3)	24 (33.8)	13 (18.3)	21 (29.6)	
*Surgical complications*						
Anastomotic insufficiency						
Yes	5 (3.5)	5 (100.0)	0	0	0	0.001
No	137 (96.5)	26 (19.0)	38 (27.7)	27 (19.7)	46 (33.6)	
Paralytic ileus						
Yes	4 (2.8)	2 (50.0)	2 (50.0)	0	0	0.217
No	138 (97.2)	29 (21.0)	36 (26.1)	27 (19.6)	46 (33.3)	
Stoma complications						
Yes	6 (4.2)	0	4 (66.7)	0	2 (33.3)	0.098
No	136 (95.8)	31 (22.8)	34 (25.0)	27 (19.9)	44 (32.3)	
Wound infection dehiscence						
Yes	23 (16.2)	4 (17.4)	5 (21.7)	3 (13.0)	11 (47.8)	0.386
No	119 (83.8)	27 (22.7)	33 (27.7)	24 (20.2)	35 (29.4)	
Space belly						
Yes	12 (8.5)	1 (8.3)	4 (33.3)	3 (25.0)	4 (33.3)	0.669
No	130 (91.5)	30 (23.1)	34 (26.1)	24 (18.5)	42 (32.3)	
Intra-abdominal abscess						
Yes	4 (2.8)	1 (25.0)	3 (75.0)	0	0	0.126
No	138 (97.2)	30 (21.7)	35 (25.4)	27 (19.6)	46 (33.3)	
*Medical complications*						
Acute renal failure						
Yes	14 (9.9)	3 (21.4)	7 (50.0)	0	4 (28.6)	0.104
No	128 (90.1)	28 (21.9)	31 (24.2)	27 (21.1)	42 (32.8)	
Pneumonia						
Yes	13 (9.2)	3 (23.1)	7 (53.8)	3 (23.1)	0	0.034
No	129 (90.8)	28 (21.7)	31 (24.0)	24 (18.6)	46 (35.7)	
*Medical complications*						
Pleural effusion						
Yes	11 (7.7)	5 (45.5)	5 (45.5)	1 (9.0)	0	0.028
No	131 (92.3)	26 (19.8)	33 (25.2)	26 (19.8)	46 (35.2)	
Arrythmia						
Yes	6 (4.2)	0	2 (33.3)	0	4 (66.7)	0.177
No	136 (95.8)	31 (22.7)	36 (26.5)	27 (19.9)	42 (30.9)	
Delir						
Yes	4 (2.8)	1 (25.0)	3 (75.0)	0	0	0.126
No	138 (97.2)	30 (21.7)	35 (25.4)	27 (19.6)	46 (33.3)	
Urinary tract infection						
Yes	11 (7.7)	3 (27.3)	3 (27.3)	1 (9.1)	4 (36.4)	
No	131 (92.3)	28 (21.4)	35 (26.7)	26 (19.8)	42 (32.1)	0.840
Multiorgan fail						
Yes	17 (12.0)	5 (29.4)	5 (29.4)	3 (17.6)	4 (23.5)	
No	125 (88.0)	26 (20.8)	33 (26.4)	24 (19.2)	42 (33.6)	0.790
*Mortality total*						
Yes	26 (18.3)	8 (30.8)	6 (23.1)	4 (15.4)	8 (30.8)	0.666
No	116 (81.7)	23 (19.8)	32 (27.6)	23 (19.8)	38 (32.8)	
*as a result of tumor progress*	9 (34.6)	3 (33.3)	3 (33.3)	1 (11.1)	2 (22.3)	0.653
*as a result of surgical complications*	6 (23.1)	3 (50.0)	0	1 (16.7)	2 (33.3)	0.217
*as a result of internal complications*	11 (42.3)	3 (27.3)	3 (27.3)	3 (27.3)	2 (18.1)	0.948

Concerning surgical complications, only anastomotic leakage showed significant difference and occurred more frequently after tumor resection than after application of a bypass anastomosis.

The analysis of postoperative medical morbidity showed a significantly increased incidence of pneumonia and pleural effusion in the group of patients with resecting procedures and with bypass anastomosis.

A total of 26 patients (28.3%) died in the hospital during treatment. In 9 cases, this was mainly caused by tumor progression. In 6 patients, surgical complications (4 × anastomotic leakage, 1 × refractory paralytic ileus and 1 × prolonged bacterial peritonitis) led to death, 11 times due to medical complications (7 × multiorgan failure, 3 × pneumonia and 1 × acute renal failure).

### Factors influencing morbidity and mortality

#### Univariate analysis

Results of all analyzed factors within the univariate analysis of 142 patients after palliative colorectal surgery are shown in Table 6.

**Table 6:** Univariate analysis of factors influencing morbidity and mortality in 142 patients after palliative colorectal surgery.

Parameters	Complications total p-Value	Complications surgical p-Value	Complications medical p-Value	Mortality p-Value
Alcohol abuse	0.004	n.s.	n.s.	0.033
Renal failure				
≥ Grade 2	0.001	n.s.	0.001	n.s.
Diabetes mellitus	0.001	n.s.	n.s.	n.s.
Pulmonary metastases	0.011	n.s.	n.s.	n.s.
Arterial hypertension	n.s.	n.s.	0.001	n.s.
Ascites	n.s.	n.s.	n.s.	0.008
Urgent surgery:				
≤6 h	n.s.	n.s.	n.s.	0.001

n.s., not significant.

The postoperative complication rate was univariately significant influenced by preexisting alcohol abuse (8 of 10 patients, 80.0% vs. 42 of 132 patients, 31.8%), renal failure stage ≧ 2 (27 of 39 patients, 69.2% vs. 23 of 103 patients, 22.3%), diabetes mellitus (26 of 42 patients, 61.9% vs. 24 of 100 patients, 24.0%) and pulmonary metastasis of colorectal cancer (16 of 29 patients, 55.2% vs. 34 of 113 patients, 30.1%).

Univariately, predictive factors for postoperative surgical morbidity were not found. However, subanalysis of individual complications showed a significant accumulation of impaired wound healing of incisions and stomata in cases with preexisting diabetes mellitus (p=0.015).

A significant increase in medical complications was seen in patients with renal failure stage ≥2 (24 of 39 patients, 61.5% vs. 22 of 103 patients, 21.4%) and arterial hypertension (37 of 91 patients, 40.6% vs. 9 of 51 patients, 17.6%). The preexisting renal failure resulted in a significant increase in postoperative acute renal (p=0.003) and multiorgan failure (p=0.019). Patients with arterial hypertension showed increased rates of pneumonia (p=0.004) and multiorgan failure (p=0.037).

Significantly more patients died in the early postoperative course in cases with preoperative evidence of ascites (17 of 60 patients, 28.3% vs. 9 of 82 patients, 11.0%) and when urgent surgery was necessary within a time interval of ≤6 h after the presentation (22 of 79 patients, 27.8% vs. 4 of 63 patients, 6.3%).

#### Multivariate analysis

In multivariate analysis, Obesity, preexisting renal failure stage ≥2, diabetes mellitus, lung metastases, age ≥75 years and alcohol abuse lead to an independent 1.3–1.8-fold increased risk for postoperative complications (see Table 7).

**Table 7:** Multivariate analysis of risk factors for morbidity and mortality in 142 palliative colorectal surgeries.

Parameters	Complications total			Complications surgical			Complications medical			Mortality		
	Odds ratio	95% CI	p-Value	Odds ratio	95% CI	p-Value	Odds ratio	95% CI	p-Value	Odds ratio	95% CI	p-Value
Obesity	1.8	0.7–3.1	0.036	n.s.			1.6	0.8–4.1	0.019	n.s.		
Renal insufficiency	1.6	0.6–3.0	0.040	n.s.			1.6	0.8–4.0	0.004	n.s.		
Diabetes mellitus	1.6	0.6–2.8	0.032	n.s.			n.s.			n.s.		
Pulmonary metastases	1.6	0.6–2.7	0.041	n.s.			n.s.			n.s.		
Age (>75 years)	1.5	0.4–2.5	0.046	n.s.			1.3	0.6–2.4	0.034	n.s.		
Alcohol abuse	1.3	0.3–2.4	0.023	n.s.			1.9	0.9–4.3	0.011	n.s.		
Arteriosclerosis	n.s.			n.s.			1.4	0.8–4.0	0.045	n.s.		
Hypertension	n.s.			n.s.			1.4	0.7–2.6	0.042	n.s.		
Urgent surgery: <6 h	n.s.			n.s.			n.s.			10.2	5.9–15.8	0.019
OP-indication: Ileus	n.s.			n.s.			n.s.			9.2	3.2–15.8	0.006
Ascites	n.s.			n.s.			n.s.			5.0	1.8–8.0	0.034

95% CI, 95% confidence interval; n.s., not significant.

Any of the parameters did not significantly influence the risk of surgical morbidity.

A multivariate significant increase in medical morbidity after surgery resulted from obesity, preexisting renal failure stage ≥2, advanced age (≥75 years), alcohol abuse, arteriosclerosis and arterial hypertension (odd ratios between 1.3 and 1.9).

The most significant factors influencing postoperative mortality were, therefore, an urgent surgical intervention within a time interval of ≤6 h after the presentation, which increased the risk of early postoperative death of patients to 10.2-fold, an existing ileus to 9.2-fold and ascites to 5.0-fold.

## Discussion

Within surgical oncology, palliative surgery offers an effective instrument for controlling and reducing disease-specific symptoms and thus improving the quality of life in the terminal period.

Unfortunately, strategies for the effective use of palliative surgical procedures in colorectal cancer cannot be standardized.

We initiated the present study as a contribution to individualized therapy in a situation of incurable colorectal carcinoma disease. A structured analysis of complications and mortality after surgery was carried out as well as an examination of the potential influence of existing concomitant conditions, tumor-associated complaints, parameters of malignancies and factors determining the indication for surgery.

The analysis of the demographic data showed a multivariate significant influence of the age of the patients of ≥75 years on the overall and medical morbidity. Especially in elderly patients, increased peri- and postoperative complications must be expected due to a limitation of the physiological reserve, and a higher incidence of comorbidities, irrespective of the underlying oncological disease [4]. The results of the present study confirm that this is also valid for patients who need to undergo palliative colorectal surgery.

The primary importance of comorbidities for the early postoperative course was confirmed by the differentiated analysis of existing concomitant diseases in palliative patients. Thus, a statistically relevant influence of obesity, renal insufficiency in stage 2 and above, arteriosclerosis, arterial hypertension, diabetes mellitus and alcohol abuse on the total and/or internal complication rate was demonstrated.

After surgery of obese patients with colorectal cancer, complications can reach rates of up to 65% and occur preferentially in the case of synchronously existing further comorbidities [5], [6]. Up to 18% of cases are lethal [7]. According to the results of our series, a palliative treatment approach does not necessarily lead to a further deterioration of the early postoperative prognosis. We found similar results with a morbidity rate of 61% and a mortality rate of 16.7% for obese patients. Similar to studies on patients with curative treatment approaches, we confirmed obesity as a risk factor for the complication rate overall and regarding medical complications.

Preexisting renal insufficiency predisposes to a large number of postoperative complications. These include wound healing disorders, pneumonia, urinary tract infections, acute renal failure, cardiac events and septic courses with multiorgan failure [8], [9]. Analogous correlations were found analyzing complications in the cohort of patients in this study. We showed a significant correlation to the occurrence of acute renal (p=0.003) and multiorgan failure (p=0.019).

Overall, the risk of complications after colorectal surgery is up to 2.4 times higher due to preexisting renal failure [10]. In the present analysis, an odd ratio of 1.6 was determined. In large series, lethality rates reach levels of 13.3% [11], [12], [13]. In our study, these figures were significantly exceeded, with a mortality rate of 25.6%. Based on the results obtained in palliative patients with preoperative restriction of renal function, a significant increase in mortality must be expected. Despite the potentially increased risk of mortality, patients with preexisting renal dysfunction should not be excluded from palliative surgery. However, they do require more intensive monitoring. There are only a comparatively small number of studies that examine the significance of high blood pressure for the early postoperative course after colorectal surgery. Nonsurgical complications such as renal, cardiac and pulmonary events are described after operations with curative intent [8], [14]. The present study in palliative patients also showed a hypertension-induced multivariate significant increase in postoperative medical morbidity to 1.4 times. When we examined individual complications, we noted a statistically relevant increase in cases of pneumonia (p=0.004) and multiorgan failures (p=0.037).

Diabetes mellitus induces wound healing disorders after surgery [15]. Laparoscopic surgery offers advantages in this regard also for palliative patients [16]. Similarly, in our results, healing disorders of surgical incisions and stomata were significantly more frequent (p=0.015), but only 22% of all interventions performed were laparoscopic.

Furthermore, renal, pulmonary, cardiac and septic complications resulting from progressive arteriosclerosis and delay postoperative rehabilitation due to diabetes mellitus were observed and led to a 1.6-fold increased risk of complications rate [15], [17].

Preexisting alcohol abuse almost doubles morbidity and mortality rates up to 46 and 27% after tumor surgery of the colon and rectum [18]. In this study, complications occurred in 56.3% and mortality in 30.0% of cases. Compared to the subgroup with alcohol abstinence, both were significantly more frequent (p=0.004 and p=0.033) for the early postoperative course. Hence, the increased complication and mortality risk of patients with alcohol abuse after potentially curative colorectal surgery is, according to the present analysis, also transferable to palliative interventions. The multivariate analysis confirmed an independent influence of alcohol abuse on overall morbidity with an odd ratio of 1.3 and showed a 1.9-fold increased risk of medical complications.

Cardiac insufficiency, immunosuppression, blood clotting disorders, and frequent malnutrition with corresponding vitamin and protein deficiencies of patients with alcohol abuse result primarily in disturbances of wound healing, pneumonia and even ARDS and septic disorders with multiorgan failure [19], [20], [21]. In our study, patients with excessive alcohol consumption accounted for 32% of all wound infections, 20% of all pneumonia and 30% of all cases of multiorgan failure without significant differences compared to alcohol abstinent patients.

In the group of tumor-associated characteristics, we demonstrated uni- and multivariate influence of pulmonary metastasis on the total complication rate after palliative colorectal surgery.

Pulmonary metastasis increases the risk of postoperative pneumonia or pleural effusion [22]. Although a slight accumulation of pathological intrathoracic fluid accumulation and pulmonary infections was detected, we could not confirm significant differences in this study.

In summary, none of the factors examined showed a uni- or multivariate significant influence on the overall surgical complication rate. This can be taken as an indicator for an adequate selection of patients with regard to indication and selection of the performed surgery.

In more than 50% of cases with ascites, peritoneal carcinomatosis in a further 13% excessive liver metastasis is present [23]. At this stage of the disease, the prognosis of patients is usually very limited. Mortality rates after colorectal surgery reach levels of up to 14.5% [24]. The lethal risk is increased up to 5.7-fold [25]. In comparison, an increased postoperative mortality rate of 18.3% was demonstrated in the present collection of palliatively treated patients. With an odd ratio of 5.0, the mortality risk determined was similar to the data provided in the literature studies. For this reason, operations should be limited for highly selected cases and performed as the smallest possible intervention. Accordingly, the patients analyzed were treated with the formation of a stoma in 41.6% of all cases (p=0.019).

The majority of patients with mechanical ileus needs urgent surgery.

In this situation, morbidity rate is elevated from 27 to 64% [26], [27], [28]. The mortality rate reaches up to 15.3%; in the current series, it reaches even up to 34% [29], [30]. Our analysis showed a morbidity rate of 71.4% and a mortality rate of 20.0% for patients after urgent palliative surgery for mechanical ileus. Compared to patients with a prolonged preoperative interval (complication rate 31.7%; lethality 7.3%), the outcome was significantly worse (p=0.005 and p=0.078). Even the detection of an ileus without further symptoms increased the risk of mortality by a factor of 9.2, an urgent surgical intervention by 10.2. Therefore, patients with a mechanical ileus should be conditioned using all available conservative and interventional therapies (stent, etc.) and transferred to an elective surgical indication [31], [32], [33], [34].

The results of the present study are limited in their value, mainly by the retrospective character of the study. The data acquisition stretched over a long period of 8 years in which the palliative oncological treatment of colorectal carcinoma was improved by establishing numerous effective chemotherapy protocols and biologicals. Finally, the study focused on the analysis of an influence of patient parameters, the underlying tumors, and the surgical indication on the early postoperative prognosis. Factors such as the expertise of the surgeon were not considered.

## Summary

Data supporting decisions for the surgical therapy of colorectal carcinoma in a palliative situation are still limited, and guidelines for differential management are missing. Therefore, we initiated the present study as a contribution to facilitate decision making in palliative patients.

Similar to a curative therapy approach, the study showed a marked influence of existing comorbidities of the patient on postoperative medical complications. The risk from the palliative treatment approach was not generally increased.

In the choice of the surgical procedure, resection procedures are recommended if the patient’s general condition is sufficient. In the case of local irresectability or high-risk constellations, the creation of an internal bypass or enterostomy should be preferred.

None of the analyzed parameters could be identified as a significant factor for surgical complications.

In addition to an advanced tumor with ascites, early postoperative lethality was influenced by an existing ileus and urgent surgery within 6 h after the initial presentation. Therefore, every effort should be made to convey these patients to an elective treatment approach using interventional therapy methods such as a stent or minimally invasive stoma formation.
