# Supplementary material for: Predictive factors of early outcome after palliative surgery for colorectal carcinoma
Source: Innov Surg Sci. 2020 Nov 2;5(3-4):91–103. doi: 10.1515/iss-2020-0018 (PMC8668025; doi:10.1515/iss-2020-0018)
Supplement: Supplementary file 1 [file iss-05-20200018-s001.pdf]

Reviewer Assessment

Stephan Kersting et al.: Predictive factors of early outcome after palliative surgery for colorectal carcinoma.

Reviewers' Comments to Original Submission

Reviewer 1: anonymous

Date received: 03-Aug-2020
Reviewer recommendation: **Return to author for minor modifications**
Reviewer overall scoring: **High**

Assessment Form scores: 5 = High/Yes; 3 = Medium/Adequate; 1 = Low

Is the subject area appropriate for the journal		4			
Does the title clearly reflect the paper's content?		4			
Does the abstract clearly reflect the paper's content		4			
Do the keywords clearly reflect the paper's content?		4			
Does the introduction present the problem clearly?		4			
Are the results/ conclusions justified?	5				
How comprehensive and up-to-date is the subject matter presented?		4			
How adequate is the data presentation?	5				
Are units and terminology used correctly?		4			
Is the number of cases adequate?	5				
Are the experimental methods/ clinical studies adequate?	5				
Is the length appropriate in relation to the content?		4			
Does the reader get new insights from the article?		4			
Please rate the practical significance.		4			
Please rate the accuracy of methods.		4			
Please rate the statistical evaluation and quality control.	5				
Please rate the appropriateness of the figures and tables.		4			
Please rate the appropriateness of the references.		4			
Please evaluate the writing style and use of language.		4			
Please judge the overall scientific quality of the manuscript.		4			
Are the methods used worthy of reproduction in greater deal?	Yes				
Would you be willing to review a revision of this manuscript?	Yes				

Comments to author: *In this paper the authors tried to identify risk factors for patients with colorectal cancer undergoing palliative surgery in order to facilitate a decision making process in this therapeutic setting. Therefore, a retrospective chart*

review of 142 patients was performed. All study patients underwent palliative surgery due to locally advanced, complicated or advanced metastatic colorectal cancer. In-hospital morbidity was 50.0% and mortality rate was 18.3%. While multiple independent risk factors for in-hospital morbidity was identified, emergency surgery, intestinal obstruction and ascites were the only multivariately significant parameters of in-hospital mortality. Based on their findings the authors suggest that especially in patients with intestinal obstruction, emergency surgery and/or ascites, effort should be made to transfer these patients to an elective treatment approach using interventional therapy methods such as stent or minimally invasive stoma formation.

In my opinion the authors address a very interesting clinical problem: the identification of risk factors for patients with colorectal cancer undergoing palliative surgery in order to facilitate a decision making process.
The manuscript is well designed with comprehensive statistical methods. Therefore, I would suggest just some minor changes:
1. The discussion is way too long. It should be shortened and the authors should just focus on the main clinical points.
2. The statement of the very precise summary at the end of the manuscript should also be highlighted in the abstract.

Reviewer 2: anonymous

Date received: 03-Sep-2020
Reviewer recommendation: **Return to author for major modifications**
Reviewer overall scoring: **Medium**

Assessment Form scores: 5 = High/Yes; 3 = Medium/Adequate; 1 = Low

Is the subject area appropriate for the journal	5				
Does the title clearly reflect the paper's content?	5				
Does the abstract clearly reflect the paper's content		4			
Do the keywords clearly reflect the paper's content?			3		
Does the introduction present the problem clearly?		4			
Are the results/ conclusions justified?			3		
How comprehensive and up-to-date is the subject matter presented?				2	
How adequate is the data presentation?				2	
Are units and terminology used correctly?		4			
Is the number of cases adequate?			3		
Are the experimental methods/ clinical studies adequate?		4			
Is the length appropriate in relation to the content?				2	
Does the reader get new insights from the article?			3		
Please rate the practical significance.			3		
Please rate the accuracy of methods.		4			
Please rate the statistical evaluation and quality control.			3		
Please rate the appropriateness of the figures and tables.				2	
Please rate the appropriateness of the references.	5				
Please evaluate the writing style and use of language.			3		
Please judge the overall scientific quality of the manuscript.			3		
Are the methods used worthy of reproduction in greater deal?	Yes/No				
Would you be willing to review a revision of this manuscript?	Yes/No				

Comments to author: I would propose to shorten the presentation of the data. The Reader has to fight through a huge listing of risk factors and their effect on the patients. You can see all Details in the tables. I only would present the essentials in the text and refer to the tables.
The discussion contains still a relevant amount of results, but only a few "real discussion" about the results.

Authors' Response to Reviewer Comments

Date received: 16-Sep-2020

Response to reviewer 1

Reviewer 1
In my opinion, the authors address a very interesting clinical problem: the identification of risk factors for patients with colorectal cancer undergoing palliative surgery in order to facilitate a decision making process.
The manuscript is well designed with comprehensive statistical methods. Therefore, I would suggest just some minor changes:

1. The discussion is way too long. It should be shortened and the authors should just focus on the main clinical points.
We have shortened the discussion considerably according to the recommendation of the reviewer. Among other things, the discussion no longer includes the pathophysiological effects of individual concomitant diseases of patients with regard to morbidity and mortality. Furthermore, our own results are now presented in a more focused manner in the context of the literature (see pages 12ff).

2. The statement of the very precise summary at the end of the manuscript should also be highlighted in the abstract.
We are grateful for the valuable advice of the reviewer and have included the fundamental statements of the work from the summary in the abstract (see abstract p.

Response to reviewer 2

Reviewer 2
I would propose to shorten the presentation of the data. The Reader has to fight through a huge listing of risk factors and their effect on the patients. You can see all details in the tables. I only would present the essentials in the text and refer to the tables.

At the suggestion of the reviewer, we have significantly shortened and simplified the text of the results in the revised manuscript. We have deleted references to non-significant factors. The distribution of the characteristics in the univariate analysis remained in the work because they cannot be taken from the tables. In contrast, the p-values listed in the tables have been removed. We hope this will make the work more comfortable to read and understand (see page 8ff, page 11ff).

The discussion contains still a relevant amount of results, but only a few "real discussions" about the results.
Similar criticism of the discussion was also brought up by Reviewer 1. We have now shortened the discussion considerably and concentrated more on the presentation of our own relevant influencing factors in comparison with the literature. Furthermore, based on our results, consequences for action in clinical practice have been deduced. (see page 11ff).

We would like to take this opportunity to thank you once again for the many constructive suggestions, which were a great help in the revision of the manuscript.
We are confident that our manuscript has gained quality through the extensive revision and hope that it might now be suitable for a publication in "Innovative Surgical Sciences."

Reviewers’ Comments to Revised Submission

Reviewer 1: anonymous

Date received: 26-Sep-2020
Reviewer recommendation: **Accept in present form**
Reviewer overall scoring: **High**

Assessment Form scores: 5 = High/Yes; 3 = Medium/Adequate; 1 = Low

Is the subject area appropriate for the journal		4			
Does the title clearly reflect the paper's content?		4			
Does the abstract clearly reflect the paper's content		4			
Do the keywords clearly reflect the paper's content?		4			
Does the introduction present the problem clearly?		4			
Are the results/ conclusions justified?		4			
How comprehensive and up-to-date is the subject matter presented?		4			
How adequate is the data presentation?		4			
Are units and terminology used correctly?					
Is the number of cases adequate?					
Are the experimental methods/ clinical studies adequate?		4			
Is the length appropriate in relation to the content?		4			
Does the reader get new insights from the article?		4			
Please rate the practical significance.		4			
Please rate the accuracy of methods.					
Please rate the statistical evaluation and quality control.					
Please rate the appropriateness of the figures and tables.					
Please rate the appropriateness of the references.		4			
Please evaluate the writing style and use of language.		4			
Please judge the overall scientific quality of the manuscript.		4			
Are the methods used worthy of reproduction in greater deal?	Yes				
Would you be willing to review a revision of this manuscript?	Yes				

Comments to author: Accept in present form

Reviewer 2: anonymous

Date received: 26-Sep-2020
Reviewer recommendation: **Accept in present form**
Reviewer overall scoring: **Medium**

Assessment Form scores: 5 = High/Yes; 3 = Medium/Adequate; 1 = Low

Is the subject area appropriate for the journal		4			
Does the title clearly reflect the paper's content?		4			

Does the abstract clearly reflect the paper's content		4			
Do the keywords clearly reflect the paper's content?			3		
Does the introduction present the problem clearly?		4			
Are the results/ conclusions justified?		4			
How comprehensive and up-to-date is the subject matter presented?		4			
How adequate is the data presentation?			3		
Are units and terminology used correctly?	5				
Is the number of cases adequate?		4			
Are the experimental methods/ clinical studies adequate?		4			
Is the length appropriate in relation to the content?		4			
Does the reader get new insights from the article?			3		
Please rate the practical significance.			3		
Please rate the accuracy of methods.		4			
Please rate the statistical evaluation and quality control.		4			
Please rate the appropriateness of the figures and tables.		4			
Please rate the appropriateness of the references.		4			
Please evaluate the writing style and use of language.			3		
Please judge the overall scientific quality of the manuscript.		4			
Are the methods used worthy of reproduction in greater deal?	Yes				
Would you be willing to review a revision of this manuscript?	Yes				

Comments to author: Good implementation of the proposed changes and criticism. More pleasing to read the article.
